# *Nucleospora cyclopteri* n. sp., an intranuclear microsporidian infecting wild lumpfish, *Cyclopterus lumpus* L., in Icelandic waters

**DOI:** 10.1186/1756-3305-6-49

**Published:** 2013-02-27

**Authors:** Mark A Freeman, Jacob M Kasper, Árni Kristmundsson

**Affiliations:** 1 Institute of Ocean and Earth Sciences, University of Malaya, Kuala Lumpur 50603, Malaysia; 2 Marine Research Institute, Skúlagata 4, 101, Reykjavík, Iceland; 3 BioPol, Einbúastígur 2, 545, Skagaströnd, Iceland; 4 Institute for Experimental Pathology, University of Iceland, Keldur v/Vesturlandsveg, 112, Reykjavik, Iceland

**Keywords:** Lumpfish, *Cyclopterus lumpus*, Intranuclear, Microsporidia, *Nucleospora*, Lymphocyte, Clinical signs, Kidney

## Abstract

**Background:**

Commercial fisheries of lumpfish *Cyclopterus lumpus* have been carried out in Iceland for centuries. Traditionally the most valuable part is the eggs which are harvested for use as a caviar substitute.

Previously reported parasitic infections from lumpfish include an undescribed intranuclear microsporidian associated with abnormal kidneys and mortalities in captive lumpfish in Canada. During Icelandic lumpfish fisheries in spring 2011, extensive enlargements to the kidneys were observed in some fish during processing. The aim of this study was to identify the pathogen responsible for these abnormalities.

**Methods:**

Lumpfish from the Icelandic coast were examined for the causative agent of kidney enlargement. Fish were dissected and used in histological and molecular studies.

**Results:**

Lumpfish, with various grades of clinical signs, were observed at 12 of the 43 sites sampled around Iceland. From a total of 77 fish examined, 18 had clear clinical signs, the most prominent of which was an extensive enlargement and pallor of the kidneys. The histopathology of the most severely affected fish consisted of extensive degeneration and necrosis of kidney tubules and vacuolar degeneration of the haematopoietic tissue. Intranuclear microsporidians were detected in all organs examined in fish with prominent clinical signs and most organs of apparently healthy fish using the new PCR and histological examination. One or multiple uniformly oval shaped spores measuring 3.12 ± 0.15 × 1.30 ± 0.12 μm were observed in the nucleus of affected lymphocytes and lymphocyte precursor cells. DNA sequencing provided a ribosomal DNA sequence that was strongly supported in phylogenetic analyses in a clade containing other microsporidian parasites from the Enterocytozoonidae, showing highest similarity to the intranuclear microsporidian *Nucleospora salmonis*.

**Conclusions:**

Intranuclear microsporidian infections are common in wild caught lumpfish from around the Icelandic coast. Infections can cause severe clinical signs and extensive histopathological changes, but are also present, at lower levels, in fish that do not show clinical signs. Some common features exist with the intranuclear microsporidian previously reported from captive Canadian lumpfish, but DNA sequence data is required from Canadian fish to confirm conspecificity.

Based on phylogenetic analysis and the intranuclear location of the parasite, the name *Nucleospora cyclopteri* n. sp. is proposed.

## Background

Lumpfish, or lumpsucker, *Cyclopterus lumpus*, L. 1758, are distributed throughout the North Atlantic Ocean and are a commercially important species in Greenland, Iceland, Norway, and Canada. Female lumpfish are targeted during a coastal spring fishery in Iceland as their valuable eggs are harvested for use as a caviar substitute. Juvenile lumpfish emerge from egg clutches in the spring and spend their first year of life among weed clumps in bays and fjords until they move offshore. Females and males return to the coastal regions during the spring and summer seasons to spawn [1,2].

Commercial fisheries of lumpfish in Iceland have been carried out for centuries [3] and the average annual catch the last 30 years has been around 6200 t [4]. Until recently, only the lumpfish eggs were harvested due to a lack of markets for the fish muscle. However, over the last two years this has changed due to alterations in fishing regulations that now require fisherman to bring all lumpfish caught to shore for full processing. During the processing of landed lumpfish in spring 2011, it was noticed that some fish had an extensive enlargement of the kidneys.

Captive lumpfish from Eastern Canada have previously been reported with marked pathological changes observed in the kidneys [5]. On that occasion, the pathogen responsible for the condition was an undescribed intranuclear microsporidian parasite that caused the pathology observed and also led to chronic mortalities of the tank-reared fish [5]. Microsporidians were formerly considered protists but are now classified as fungi [6]. They are spore forming organisms known to infect numerous fish species [7] which include species with intranuclear sites of infection and are also found as hyperparasites [8].

In the present study, we perform histological and molecular studies on Icelandic lumpfish and describe a novel microsporidian, *Nucleospora cyclopteri* n. sp.

## Methods

### Research material and macroscopic examination

The initial research material was sampled in July 2011 in Haganesvik in Skagafjordur in the northern part of Iceland (66° 4'34.90"N, 19° 9'22.28"W). Three fish with clinical signs in the kidney, observed by local fishermen, were sent for examination to determine an aetiology for the condition. Wet mounts of fresh kidney tissue were examined at 400× and 1000× magnification with a compound microscope and kidney samples fixed in 10% buffered formalin for histological examination and in 95% ethanol for DNA analysis.

For further examination of the nature of the disease, 10 spawning female lumpfish (length 40–48 cm) were sampled from Húnaflói Bay in northern Iceland (66° 3'37. 29"N, 20°28'18. 31"W) aboard a lumpfish vessel in April 2012. Five were selected as they showed clearly visible clinical signs in the kidney and five were selected as they appeared to be healthy with no obvious clinical signs. Immediately post mortem, each fish was dissected using sterile technique and parts of the kidneys, spleen, heart, gills, skin and eggs were fixed in 10% formalin for histological examination and 95% ethanol for molecular analysis.

During the annual Icelandic groundfish survey, performed by the Marine Research Institute in Iceland in late February and early March 2012 [9], 77 lumpfish (68 females; 33–47 cm, 9 males; 26–32 cm) from 43 sites around Iceland were randomly selected and screened for clinical signs in kidneys comparable to those formerly observed. The number of fish examined from each station ranged from a single one to nine fish.

### Histopathology

Formalin fixed samples were embedded in paraffin wax, sectioned (4 μm) and prepared for histological examination according to routine protocols. The sections were stained with either Haematoxylin & Eosin (HE) or Giemsa and screened for pathogens and histopathological changes. Furthermore, histological slides were stained with a chitin-specific stain, Calcofluor white M3R (Sigma-Aldrich), to look specifically for microsporidian spores. To evaluate the distribution and intensity of microsporidian spores in the host’s tissues, the slides were examined using a compound microscope at 400× magnification. Infections in each organ were graded as follows: (−ve) = no spores detected; (*) = light infection; less than 10 spores or a cluster of spores (inside the same cell) occasionally seen; (**) = moderate infection; spores seen in most microscopic fields, their number ranging from 10 to 100; (***) = massive infection; more than 100 spores seen in all microscopic fields. To identify with more certainty which cell types were affected, imprints of kidney were taken from fish with extensive clinical signs. These samples were air-dried, fixed in methanol and stained with Calcofluor white M3R and/or Giemsa. All tissue samples were examined at high magnification and photographs taken using a Leica DMLB compound microscope equipped with a digital camera (Leica DC300F).

### DNA sequencing and phylogenetic analyses

Dissected ethanol-preserved kidney tissues from three fish suspected to be infected with the microsporidian, and the ten fish for microsporidian screening, were further cut with a scalpel blade and approximately 25 mg of tissue digested at 56°C in tissue lysis buffer containing 100 μg/mL proteinase K until dissolved. DNA was extracted using a GeneMATRIX DNA extraction kit (EURx Poland) following the tissue protocol and 10 ng used as template DNA for PCRs. Small subunit (SSU), internal transcribed spacer (ITS) and partial large subunit (LSU) regions of the rRNA gene were amplified using previously described microsporidian primers [10,11]. An additional primer 870fwd 5^′^ tgcggcttaatttgactcaac [12] and its complementary reverse primer 870rev were used with the forward and reverse primers to allow a sufficient overlap for sequence confirmation. PCRs were performed according to the original descriptions with the use of suitable negative controls. PCR products were run alongside a 100 bp DNA ladder on 1% agarose gels, pre-stained with 1× SYBR®-safe (Invitrogen Oregon USA), and visualised with a high performance UV transilluminator (UVP, Cambridge UK). Direct sequencing reactions were done using BigDyeTM Terminator Cycle Sequencing chemistry utilising the same primers. DNA sequencing was performed in both directions on all positive PCR products, of the expected sizes, and compared to sequences available in the GenBank databases using nucleotide-nucleotide BLAST searches [13] to verify a microsporidian origin. The contiguous sequence was obtained manually using CLUSTAL-X [14] and BioEdit [15]. CLUSTAL X was used for the initial sequence alignments, with the settings for gap opening/extension penalties being adjusted manually to achieve optimum alignments, and manually edited using the BioEdit sequence alignment editor. Percentage divergence matrices were constructed in CLUSTAL X using the neighbour-joining method based on the Kimura 2-parameter model [16]. Microsporidia from the Enterocytozoonidae are typically assigned as new species or given generic status partly based on SSU rDNA sequence data [17]. Therefore, alignment files of 16 taxa, representative of microsporidia from the family Enterocytozoonidae and related basal taxa, consisting of 1,269 characters of SSU sequence data were used in the phylogenetic analyses.

Phylogenetic analyses were performed using the maximum likelihood methodology in PhyML [18] with the general time-reversible substitution model selected and 1000 bootstrap repeats, and Bayesian inference (BI) analysis using MrBayes v. 3.0 [19]. For the BI analysis, models of nucleotide substitution were first evaluated for the alignment using MrModeltest v. 2.2 [20]. The most parameter-rich evolutionary model based on the AIC was the general time-reversible, GTR + I + G model of evolution. Therefore, the settings used for the analysis were nst = 6, with the gamma-distributed rate variation across sites and a proportion of invariable sites (rates = invgamma). The priors on state frequency were left at the default setting (Prset statefreqpr = dirichlet (1, 1, 1, 1). Posterior probability distributions were generated using the Markov Chain Monte Carlo (MCMC) method with four chains being run simultaneously for 1000.000 generations. Burn in was set at 2.500 and trees were sampled every 100 generations making a total of 7.500 trees used to compile the majority rule consensus trees.

### Development of nested PCR

DNA extracted from the three fish initially sampled was used to develop the nested PCR. Four controls were used in the assay development to monitor for contamination: a template blank containing no DNA, a negative fish tissue, a positive sample for *Desmozoon lepeophtherii*[8], which was to be positive in the first round, but negative in the second round of the nested PCR and a positive control for *N*. *cyclopteri* from the initial DNA extractions in this study. The first pair of primers, LN1-fwd 5^′^ atcctaggatcaaggacgaag and LN1-rev 5^′^ aatgatatgcttaagttcagg were modified from those used by Freeman *et al*. [8] and were designed to be semi-specific for microsporidians from the genus *Nucleospora* amplifying a 950 bp region of the SSU and ITS region. A second specific primer pair LN2-fwd 5^′^ ctgcttaatttgactcaacgc and LN2-rev 5^′^ tactgctcctcaaatagtatg targets a 590 bp region, within the first 950 bp amplicon, covering partial SSU and partial ITS regions of the gene, and used 1 μl of the PCR products from round one as a DNA template. Both sets of primers use the same PCR profile: initial denaturing for 4 min at 95°C followed by 35 cycles of: 94°C 30 s, 55°C 45 s, 72°C 1 min, with a terminal extension at 72°C for 7 min. PCR products were run alongside a 100 bp DNA ladder on 1% agarose gels, pre-stained with 1× SYBR®-safe (Invitrogen, Oregon USA), and visualised with a high performance UV transilluminator (UVP, Cambridge UK). Positive PCR bands from all fish (either first or second round PCR) were purified using a GeneMATRIX PCR clean up kit (EURx Poland) and sent for direct sequencing, in both directions, using the same primers from the positive amplifications.

In accordance with section 8.6 of the ICZN’s International Code of Zoological Nomenclature, details of the new species have been submitted to ZooBank with the life science identifier (LSID) zoobank.org:pub:2659C0D4-ADF1-44E5-A53A-C7507B64D0BC.

## Results

### Clinical signs, distribution and prevalence of infected fish

**Figure 1:**
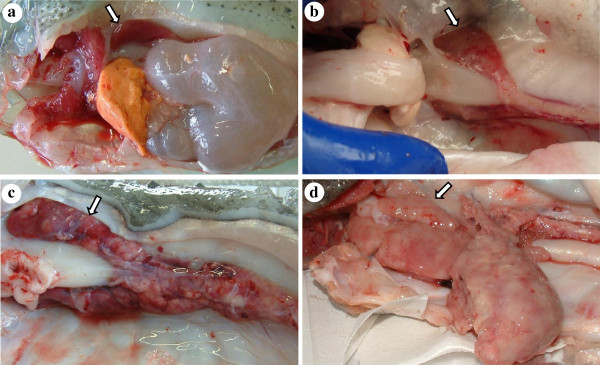
**Clinical signs of infection in the kidney of lumpfish, *Cyclopterus lumpus*, caught in Icelandic waters (arrowed).** (**a**) Normal kidney. (**b**) Mild clinical signs, patchy pallor of kidney. (**c**) Marked renomegaly with extensive pallor. (**d**) Extensive widespread enlargement and pallor of the entire kidney.

The most prominent clinical signs were observed in the kidneys. The extent of clinical signs varied considerably between individual fish. Some had normally sized kidneys which had a patchy pallor compared to smooth and evenly red normal kidney (Figure 1a, b). Other fish had various degree of renomegaly and kidney pallor. In the most severe cases the kidneys were extensively and uniformly enlarged, had a whitish appearance and apparently little or no normal tissue visible (Figure 1c, d). Fish with noticeable renomegaly, occasionally showed other clinical signs such as exophthalmos and small lesions on the skin.

**Figure 2:**
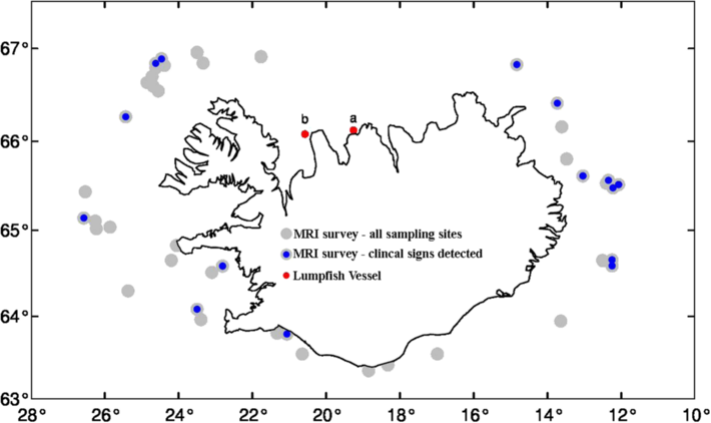
**Sampling location off the Icelandic coast.** Grey dots represent the sites where lumpfish were sampled in the MRI survey; blue dots the sites where fish with visible clinical signs in the kidney were observed. The two red dots show: (**a**) the location of the initial three fish sent for examination in spring 2011 and (**b**) the site where the 10 fish were collected in spring 2012 for thorough examination; 5 with clinical signs and 5 apparently healthy fish.

Lumpfish, with various grades of clinical signs in the kidney, were observed at 12 of the 43 sites when fish randomly collected aboard the bottom trawl survey were examined (Figure 2). Furthermore, of all the 77 fish examined, 18 had clinical signs, of which 17 were female and one was male. Mostly there was one fish at each station, however, in some cases, multiple fish from certain sites showed clinical signs, for example four of five fish sampled at one site and two of two sampled fish at another site.

### Histopathology

**Figure 3:**
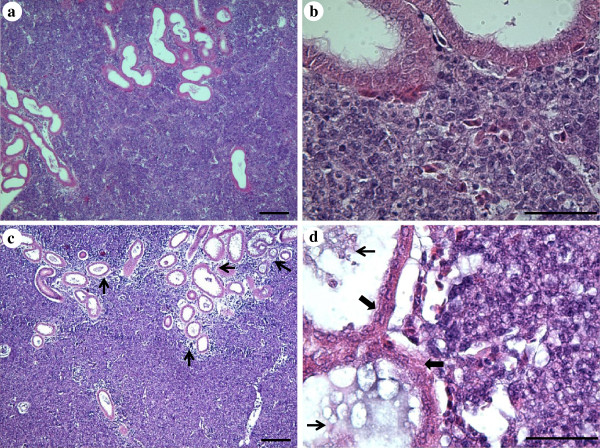
**Haematoxylin and Eosin stained histological sections of kidney tissues with varying degrees of infection.** Lightly infected kidney with no apparent pathological changes (**a**) and (**b**), heavily infected kidney with severe pathological changes (**c**) and (**d**). Note the obvious necrotic areas around kidney tubules (thin arrow) (**c**) and necrosis of tubular epithelial cells (broad arrow) with protein cast and necrotic cell debris within tubules (thin arrow) (**d**). Scale bar = 100 μm.

**Figure 4:**
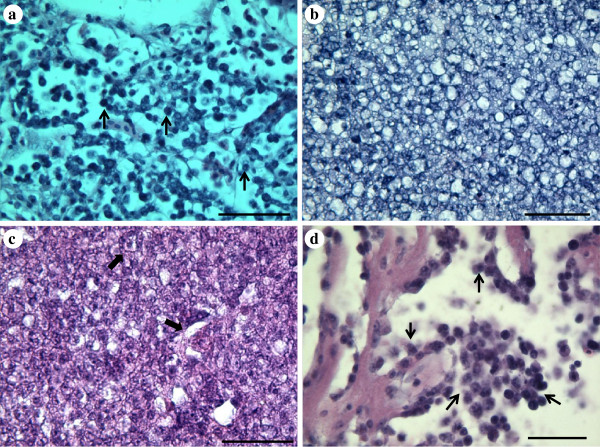
**Histological tissue sections from very heavily infected fish.** (**a**) Severe necrosis of renal haematopoietic tissue with lymphocyte infiltration (arrow). (**b**) Vacuolar degeneration of haematopoietic tissue of the kidney. (**c**) Area with collapsed kidney tubules (broad arrow). (**d**) Myocardial inflammation showing infiltration of lymphocytes (arrow). (**a**) and (**b**) Giemsa stain. (**c**) and (**d**) Haematoxylin and Eosin stain. Scale bars = 100 μm.

No obvious histopathological changes were observed in fish showing no clinical signs (Figure 3a, b). Moderate to severe tissue changes were, however, observed in all fish showing clinical signs of disease, which were most prominent in the kidney. These changes were dominated by degeneration and necrosis of tubules (Figure 3c) and glomeruli. Many tubules contained protein cast and necrotic cell debris of the epithelial layer (Figure 3d), with subsequent collapse of those tubuli worst affected. Vacuolar degeneration of haematopoietic tissue cells was frequent and necrotic foci were observed with marked lymphocyte infiltration (Figure 4a, b, c). In the most severely infected fish, large areas of the kidney were almost totally necrotised, with massive numbers of microsporidian spores surrounded by necrotic cell debris. An inflammatory response with infiltration of lymphocytes was also evident in other organs such as the heart (Figure 4d).

### *Nucleospora cyclopteri* and affected cells

Examination of the initial three lumpfish sampled from Haganesvik in Skagafjordur in July 2011, confirmed heavy microsporidian infections in their kidneys. Examination of wet mounts, revealed oval shaped microsporidian spores, their length ranging from 2.9–3.5 μm (mean ± stdev = 3.12 ± 0.15) and their width 1.1–1.5 μm (1.30 ± 0.12) (n = 30).

**Figure 5:**
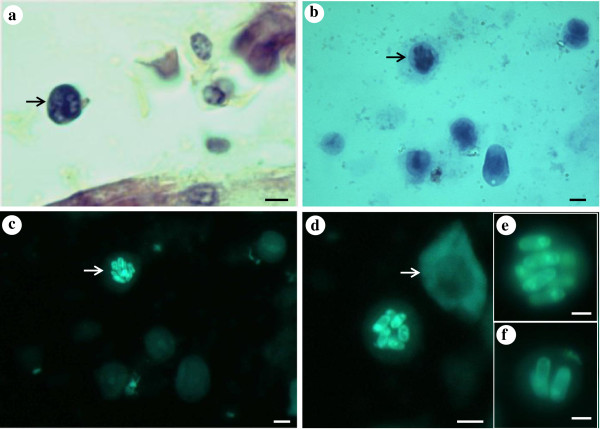
**Microsporidian infected lymphocytes stained with Giemsa (a and b) and Calcofluor white (c-f).** (**a**) Histological section showing a lymphocyte from a heart infected with numerous microsporidian spores (arrow). (**b**–**f**) Kidney imprints; (**b** and **c**) Lymphocyte showing at least 10 microsporidian spores inside a lymphocyte. (**d**) A lymphocyte infected with numerous spores that occupy most of the nucleus; note the size of infected cell compared to erythrocyte (arrow). (**e** and **f**) Higher magnification of microsporidian spores inside the nucleus of affected cells. Scale bar: (**a**)–(**d**) = 4 μm; (**e**), (**f**) = 2 μm.

Affected cells had all morphological characteristics of lymphocytes and lymphocyte precursor cells. They were round shaped, 8–12 μm in diameter, had a large nucleus occupying most of the cell, a compact chromatin and a thin rim of basophilic cytoplasm which stained sky blue in Giemsa. The number of microsporidian spores inside a single cell nucleus ranged from 1 – 14 (Figure 5a-f).

### The presence of *Nucleospora cyclopteri* in different host organs

**Figure 6:**
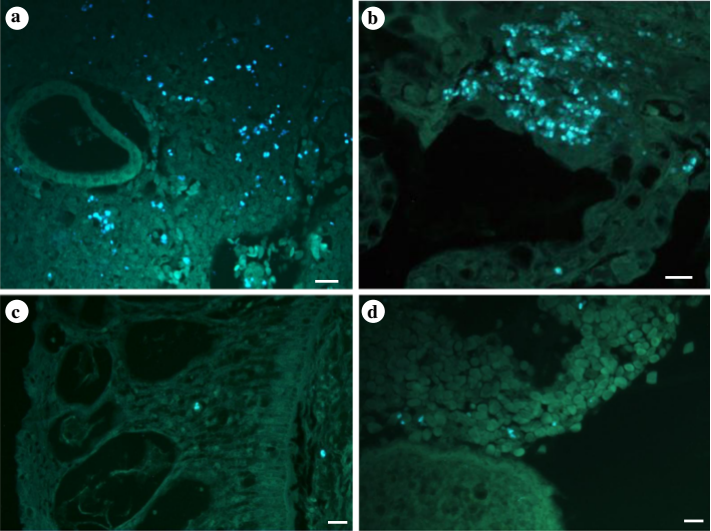
**Tissue sections stained with Calcofluor white showing numerous microsporidian spores inside affected cells within kidney, gills, skin and ovary tissues.** (**a**) Heavily infected kidney showing numerous microsporidian spores distributed in the parenchyma. (**b**) Massive infection of microsporidian spores in the primary lamellae of the gills. (**c**) Microsporidian spores in the skin epidermis and the basal membrane. (**d**) Spores within the ovarian stroma. Scale bars = 20 μm.

Microsporidian spores were found in nine of the 10 female lumpfish sampled in April 2012 using fluorescence stained histological sections; four of the apparently healthy ones (fish 1–5) and all five that had clinical signs (fish 6–10). Infections were found in all organs examined in the fish with clinical signs; kidneys, spleen, gills and heart being most heavily infected but lighter infections in the skin and the ovary (Table 1). In the kidneys and the spleen the spores were distributed in the parenchyma (Figure 6a). In the heart, the spores were most commonly detected in the myocardium, however, in the heaviest infections they also extended into the epicardium. The gills were usually heavily infected with numerous spores seen; most frequently in the primary lamellae but extending somewhat into the secondary lamellae in severely infected fish (Figure 6b). Relatively few spores were seen in the skin and the ovary but some numbers of spores were normally detected in the dermal and epidermal layers of the skin and also in the ovarian stroma (Figure 6c, d). In the four apparently healthy, but infected fish, the infections were light and spores, or clusters of spores within the same cell nucleus, were occasionally detected in renal tissue, splenic tissue and the myocardium of the heart. No spores were detected in the skin, the gills or the ovary of these fish.

**Table 1:** Results of microsporidian screening of selected organs with nested PCR and Calcofluor white stained histological sections

	**Clinical signs**	**Fish lenght (cm)**	**Method**	**Kidney**	**Heart**	**Spleen**	**Skin**	**Gills**	**Eggs**
**Fish 1**	**None**	**44**	**Fluorescence**	**-ve**	**-ve**	**-ve**	**-ve**	**-ve**	**-ve**
			**PCR 1**	**-ve**	**-ve**	**-ve**	**-ve**	**n.s.**	**-ve**
			**PCR 2**	**-ve**	**+**	**++**	**-ve**	**n.s.**	**++**
**Fish 2**	**Subclinical**	**41**	**Fluorescence**	*	*	*	**n.s.**	**n.s.**	**-ve**
			**PCR 1**	**+**	**+**	**-ve**	**-ve**	**n.s.**	**-ve**
			**PCR 2**	**++**	**++**	**++**	**++**	**n.s.**	**++**
**Fish 3**	**Subclinical**	**40**	**Fluorescence**	*	*	*	**-ve**	**-ve**	**-ve**
			**PCR 1**	**++**	**++**	**+**	**-ve**	**n.s.**	**-ve**
			**PCR 2**	**n.a.**	**n.a.**	**++**	**++**	**n.s.**	**++**
**Fish 4**	**Subclinical**	**41**	**Fluorescence**	*	*	**-ve**	**-ve**	**-ve**	**-ve**
			**PCR 1**	**-ve**	**+**	**+**	**+**	**+**	**+**
			**PCR 2**	**++**	**++**	**++**	**++**	**++**	**++**
**Fish 5**	**Subclinical**	**41**	**Fluorescence**	*	*	**-ve**	**-ve**	**-ve**	**-ve**
			**PCR 1**	**+**	**-ve**	**-ve**	**-ve**	**+**	**+**
			**PCR 2**	**++**	**++**	**+**	**++**	**++**	**++**
**Fish 6**	**Macroscopic**	**48**	**Fluorescence**	**	*	*	*	**	*
			**PCR 1**	**++**	**++**	**n.s.**	**++**	**-ve**	**-ve**
			**PCR 2**	**n.a**	**n.a.**	**n.s.**	**n.a.**	**++**	**++**
**Fish 7**	**Macroscopic**	**42**	**Fluorescence**	***	**	***	*	**	*
			**PCR 1**	**++**	**++**	**n.s.**	**++**	**+**	**+**
			**PCR 2**	**n.a.**	**n.a.**	**n.s.**	**n.a.**	**++**	**++**
**Fish 8**	**Macroscopic**	**43**	**Fluorescence**	**	**	**	*	*	*
			**PCR 1**	**++**	**++**	**-ve**	**++**	**++**	**++**
			**PCR 2**	**n.a.**	**n.a.**	**++**	**n.a.**	**n.a.**	**n.a.**
**Fish 9**	**Macroscopic**	**40**	**Fluorescence**	***	**	***	*	***	*
			**PCR 1**	**++**	**++**	**++**	**++**	**++**	**-ve**
			**PCR 2**	**n.a.**	**n.a.**	**n.a.**	**n.a.**	**n.a**	**-ve**
**Fish 10**	**Macroscopic**	**44**	**Fluorescence**	***	**	***	*	***	**-ve**
			**PCR 1**	**++**	**++**	**++**	**+**	**n.s.**	**+**
			**PCR 2**	**n.a.**	**n.a.**	**n.a.**	**++**	**n.s.**	**++**

Five were apparently healthy (fish 1–5) and five had obvious clinical signs (fish 6–10).

Fluorescence: -ve = (−ve) = no spores detected; * = light infection; less than 10 spores or a cluster of spores (inside the same cell) occasionally seen; ** = moderate infection; spores seen in most microscopic fields, their number ranging from 10 to 100; *** = massive infection; more than 100 spores seen in all microscopic fields.

PCR: - ve = No visible band; + = Faint band visible; ++ = Clear to strong band; n.a. = Not applicable (if clearly positive (++) in round 1 the second round was not performed). Other abbreviation: n.s. = No sample.

All five fish showing clinical signs of microsporidiosis (fish 6–10) were strongly positive using the first round of the PCR in one or more tissues (Table 1). Tissues that were consistently positive in round one were kidney and heart, followed by skin, gills and spleen. The eggs were not strongly positive in any of the five positive fish in round one. All weakly positive or negative tissues from round one were confirmed as positively infected using the second round PCR, except for the eggs in fish nine which remained negative. Fish with no clinical signs (fish 1–5) of infection were either negative or weakly positive in round one, with the exception of fish 3 that was positive in the heart and kidney tissues. All fish (1–5) were shown to be positive for the microsporidian in the second round of the PCR. Some positive PCR products from all ten fish were purified and sequenced to confirm the correct microsporidian sequence had been amplified.

### DNA sequencing and phylogenetic analyses

A contiguous microsporidian DNA sequence of 1869 bp of the rRNA gene, including partial SSU (1182 bp), complete ITS region (361 bp) and partial LSU (326 bp), was generated from the three fish with abnormal kidneys from Haganesvik in Skagafjordur. There was no intraspecific variation found between sequences generated from the three different fish when sequencing the PCR product directly. In addition, partial rRNA gene sequences were obtained for the 10 fish used in the nested PCR. If the fish were positive in the first round of the PCR then those products were sequenced (950 bp), if positive in the second round then those products were sequenced (590 bp). No intraspecific differences between sequences were observed between any fish.

The sequence has been submitted to GenBank with the accession number [KC203457]. A BLAST search of the sequence in the databases revealed a 96% similarity to isolates of *Nucleospora salmonis*, an intranuclear microsporidian infecting salmonid fishes worldwide [21].

A percentage identity matrix generated for 11 related taxa within the Enterocytozoonidae demonstrates the genetic distances that might be expected at the species level for this group (Table 2). There is 99.1–99.84% similarity between different isolates of *N*. *salmonis* amplified from different host fish species. There is an 86.08% and 91.18% similarity between *N*. *salmonis* from Atlantic salmon and two unidentified *Nucleospora* sp., the similarity between *N*. *cyclopteri* and the same two *Nucleospora* spp. is 85.32 and 90.67% respectively. The genus *Desmozoon* is 87.32% and 87.13% similar to *N*. *cyclopteri* and *N*. *salmonis* from Atlantic salmon. Other more basal genera from the group, such as *Hepatospora* are more distantly related (Table 2).

**Table 2:** Percentage divergence of SSU rDNA sequences for selected members of the Enterocytozoonidae

	**1.**	**2.**	**3.**	**4.**	**5.**	**6.**	**7.**	**8.**	**9.**	**10.**	**11.**
**1.***Nucleospora cyclopteri*		96.14	96.4	96.01	96.56	90.67	85.32	84.62	85.09	87.32	79.37
**2.***Nucleospora salmonis* (AS)	1219		99.51	99.1	99.67	91.18	86.08	85.99	85.7	87.13	79.56
**3.***Nucleospora salmonis* (CS)	1249	1218		99.35	99.84	91.41	86.17	86.12	86.05	87.2	79.66
**4.***Nucleospora salmonis* (AH)	776	775	775		99.48	89.65	83.33	86.24	84.98	85.36	78.65
**5.***Nucleospora salmonis* (RT)	1249	1218	1248	776		91.5	86.33	86.26	86.07	87.3	79.79
**6.***Nucleospora* sp. eel	1190	1157	1187	773	1188		86.21	84.06	84.47	87.95	79.96
**7.***Nucleospora* sp. (ES)	1247	1214	1244	774	1244	1189		80.18	85.31	91.09	75.89
**8.***Enterospora hepatopenaei*	780	778	778	734	779	778	782		89.71	94.36	79.27
**9.***Enterospora canceri*	805	804	803	506	804	747	803	554		84.87	77.23
**10.***Desmozoon lepeophtherii*	1222	1189	1219	772	1220	1187	1223	780	780		77.31
**11.***Hepatospora eriocheir*	955	954	954	768	955	953	954	772	549	952	

Percentage similarity above the diagonal and number of bases of SSU rDNA analysed below the diagonal.

AH = Atlantic halibut; AS = Atlantic salmon; CS = chinook salmon; ES = English sole; RT = rainbow trout.

**Figure 7:**
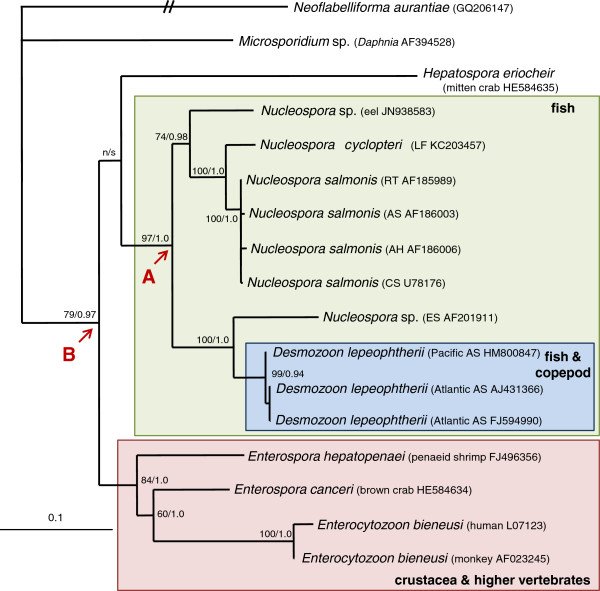
**Maximum likelihood phylogeny of the Enterocytozoonidae based on 1269 characters of aligned SSU rDNA sequence data.** Maximum likelihood phylogeny of the Enterocytozoonidae based on 1269 characters of aligned SSU rDNA sequence data. Node A gives rise to a robustly supported clade (green box) that contains intranuclear microsporidian parasites of fish, some of which are known to also infect caligid copepods (blue box); *Hepatospora eriocheir*, forms an unsupported branch at the base of the fish clade. The sister clade to the fish-infecting group (red box) contains other genera isolated from crustacea and also higher vertebrates, and is well-supported from node B. Numbers at the nodes represent branching support using non-parametric bootstraping (ML 1000 replications) and Bayesian posterior probabilities, support values <50 for ML and <0.95 for Bayesian analyses are considered not supported (n/s). Fish abbreviations and accession numbers given in parentheses: LF lumpfish, CS chinook salmon, AH Atlantic halibut, RT rainbow trout, AS Atlantic salmon, ES English sole. *Neoflabelliforma aurantiae* is used as an outgroup and to root the tree.

Phylogenetic analyses, of the Enterocytozoonidae based on 1269 characters of aligned SSU rDNA sequence data, using maximum likelihood Bayesian methodologies produced congruent trees topologies (Figure 7). The sequence for *N*. *cyclopteri* was robustly supported in a clade that contained other intranuclear microsporidian parasites of fish from the genus *Nucleospora*.

## Discussion

*Nucleospora cyclopteri* is a common microsporidian parasite infecting lumpfish around Iceland. Fish with visible clinical signs, like those infected with *N*. *cyclopteri*, were found at 12 of 43 sites around the country. Although all fish were not screened for the microsporidian, the cause of the clinical signs observed is assumed to be *N*. *cyclopteri* as all other fish screened with equivalent signs were infected with this microsporidian. Even though the prevalence data are limited to a few fish examined at each sampling site, the geographic distribution is wide. It causes severe clinical signs and extensive histopathological changes.

This is the first report of intranuclear microsporidiosis in wild lumpfish, *Cyclopterus lumpus*. However, a microsporidian infection has previously been reported from lumpfish reared in a recirculation facility in Canada, which was associated with chronic fish mortalities [5]. That paper describes the clinical, histopathological and ultrastructural features of an intranuclear microsporidian. Mullins *et al*. [5] suggested that the lumpfish microsporidian they found belonged to the genus *Enterocytozoon* based on similarities to the previously described species *Enterocytozoon bieneusi* and *E*. *salmonis* (syn. *Nucleospora salmonis*), both with regard to morphology and the type of immune response, but they did not make a formal species description. The former species is a non-intranuclear intestinal parasite of humans and numerous higher vertebrates [22,23], the latter is a well documented intranuclear microsporidian pathogen which has been identified in various salmonid species [24,25] and also from Atlantic halibut, *Hippoglossus hippoglossus*[26].

Phylogenetic analyses (Figure 7) and a percentage identity matrix (Table 2) show that *N*. *cyclopteri* is most closely related to isolates of *N*. *salmonis* with an identity of approximately 96% to all isolates [21]. Intraspecific variation of *N*. *salmonis* between four separate isolates, amplified from different host fish, is between 99.1–99.84% (Table 2), indicating that a similarity of 96% would represent a different species and not another isolate of *N*. *salmonis*. In addition to this, the spore size of 3.12 × 1.30 μm and evenly oval shape in *N*. *cyclopteri* is untypical for the family group that generally have smaller more ovoid to rounded spores with sizes recorded for *Nucleospora* spp. of 1.6 × 0.8 μm and 2.0 × 1.0 μm; *Enterocytozoon* spp. of 1.5 × 0.8 μm and 1.1 × 0.7 μm; *Enterospora* 1.3 × 0.7 μm and *Desmozoon* 2.84 × 1.83 μm [27].

Comparing the microsporidian infection described in this paper to the one from Canada [5], some similarities are evident; macroscopic clinical signs include renomegaly and exophthalmos, lymphocytes and lymphoblasts are the target cells in both cases and infections are detected in all organs examined, via vascular migration of infected cells. Other things are more difficult to compare, such as the histopathology and the morphology and size of the microsporidian spores. The histopathological description made by Mullins *et al*. [5] is quite brief; mainly describing an infiltration of lymphocyte like cells, morphological features of the target cells and their presence in all organs examined, but no actual tissue damage associated with infections. Histopathological changes observed in the lumpfish in the present paper were in many cases considerable including extensive degeneration and necrosis of kidney tubules and vacuolar degeneration of the haematopoietic tissue. It seems that a lymphocytotsis or lymphoblastosis type of inflammatory reaction, with a subsequent prominent hyperplasia, and affected cells being lymphocytes or lymphocyte precursor cells, is a relatively common feature of intranuclear microsporidiosis [28-31]. Except for one fish, all the 13 fish, which where thoroughly examined in the present study, had either light subclinical infections or severe infection with considerable or extreme clinical signs. As the extent of the clinical signs of the fish examined by Mullins *et al*. [5], such as renomegaly, is not obvious, it is possible that these fish had lighter infections and hence lacked severe histopathological changes, which might occur at later stages of infection in wild fish. Comparing the spore morphology observed in these two studies, the shape is very similar, i.e. uniformly oval and not tapered at one end, which is a common feature of some microsporidian spores. However, the spore size is considerably different, i.e. 2.1 μm x 1.0 μm in the previous study compared to a mean size of 3.12 μm x 1.30 μm in the present study. Mullins *et al*. [5] made spore measurements using ultrathin sections on a TEM but fresh spores were used in the present study. Hence, the spores from the Canadian lumpfish had been fixed in glutaraldehyde and processed for TEM and might have shrunk during this procedure. Furthermore, when using ultrathin sections, one cannot be sure that the plane of section is through the entire length and width of the spore. Consequently, the spore size in these two studies is not suitable for direct comparison and fresh spores from Canadian lumpfish should be analysed. Although some similarities are apparent between the microsporidians described in these two studies, DNA sequence data is required from Canadian lumpfish to confirm their conspecificity.

Currently, the life cycle of *Nucleospora cyclopteri* and its route of infection/transmission is not known. However, many microsporidians infecting fish have a simple direct life cycle and no vector or intermediate host is required [32]. This simple form of direct transmission between fish is known to occur in the congener *Nucleospora salmonis*, which is the closest known relative to *N*. *cyclopteri* in our phylogenetic analyses. Indeed, from our histology and PCR results, we found that the kidney and gills were very common sites of infection, indicating that these organs could be the sites for direct transmission to occur, being excreted from the kidney and entering another fish host via the gills.

Another close relative, and the robustly supported sister taxon in the phylogenetic tree, *Desmozoon lepeophtherii*, is a hyperparasite of the salmon louse *Lepeophtheirus salmonis* and also an intranuclear parasite of Atlantic salmon [27,33,34]. It is possible that multiple hosts or a complicated life cycle could occur in *N*. *cyclopteri* infections in lumpfish. Several things could support this: 1) According to Heuch *et al*. [35] lumpfish are a preferred host for the parasitic copepod *Caligus elongatus* and might serve as a reservoir for caligid infections of other fish species. 2) One of the clinical signs detected in our study were small lesions on the skin similar to those caused by parasitic copepods. 3) Histological examination revealed microsporidian spores in the most outer layers of lumpfish skin. At present this remains speculative, and additional studies are needed to examine whether *C*. *elongatus* are infected with *N*. *cyclopteri* and how transmission of the parasite might take place. In the present study spores of *N*. *cyclopteri* were also found in close association with the eggs suggesting that spores may also be vertically transmitted. Some microsporidia that infect crustaceans are known to be transovarially (vertically) transmitted [36,37] and some pathogens that enter the microphyle of the egg before its shell fully matures are known to cause bacterial infection such as *Renibacterium salmoninarum*[38]. Screening larval lumpfish soon after hatching, using the nested PCR developed in this study, would be a simple method to check for the presence of vertical transmission as a route of infection for this microsporidian. It will become necessary to evaluate such routes of infection for important pathogens of lumpfish, in order to help limit their spread and impact in captive fish. Lumpfish are becoming increasingly more important for the aquaculture industry, being used as cleaner fish in Atlantic salmon cages for the removal of salmon lice. However, their culture is problematic as they are susceptible to numerous pathogens [39].

The extreme pathology seen in some fish, which looks highly unlikely to be reversible, raises the question whether infections could have an impact upon lumpfish populations. More complete data on prevalence and severity of infections, with regard to fish age, geographic distribution and time of year, are required to determine any potential mortality rates.

### Taxonomic summary

Phylum: Microsporidia (Balbiani, 1882)

Class: Microsporea (Levine & Corliss, 1963)

Order: Microsporida (Balbiani, 1882)

Family: Enterocytozoonidae (Cali & Owen, 1990)

### Specific diagnosis

Fresh spores are evenly oval in shape, length 2.9–3.5 μm width 1.1–1.5 μm, with two lines of symmetry being evenly rounded at both ends. Mature spores, numbering between 1–14, are found inside lymphocyte nuclei in haematopoietic and other tissues. SSU rDNA sequence data confirms a close association with other *Nucleospora* species and robustly places *N*. *cyclopteri* within the Enterocytozoonidae.

Type host: Atlantic lumpfish (*Cyclopterus lumpus*, L. 1758)

Location: Coastal waters of Iceland

Type location: Skagafjordur, northern Iceland (66° 4'34.90"N, 19° 9'22.28"W).

Site of infection: The nucleus of lymphocytes and lymphocyte precursor cells. Infected lymphocytes infiltrate haematopoietic tissues (kidney and spleen), heart myocardium, gills and skin.

Etymology: The specific name *cyclopteri* refers to the generic assignment of the host fish.

Type material: Two stained wet mount slides and two histological sections have been submitted to the collections of the Natural History Museum, London, and assigned the accession numbers: (kidney tissue) NHMUK 2013.1.15.1, (heart tissue) NHMUK 2013.1.15.2, (kidney imprint) NHMUK 2013.1.15.3, (kidney imprint) NHMUK 2013.1.15.4. DNA sequence data has been submitted to GenBank with the accession number [KC203457].

## Conclusions

We have described a novel intranuclear microsporidian, *Nucleospora cyclopteri*, infecting the nuclei of lymphocyte and lymphocyte precursor cells in lumpfish *Cyclopterus lumpus*. Gross clinical signs of infection, notably severe inflammation of the kidney, were found at numerous locations around the Icelandic coast with a prevalence of 23% (18/77 fish). However, using the new nested PCR, infection rates were shown to be significantly higher and could reach 100%. Marked histopathological changes were evident in fish with clinical signs that are unlikely to be reversible, indicating that *N*. *cyclopteri* may have a negative impact on spawning lumpfish populations. *Nucleospora cyclopteri* is phylogenetically related to other intranuclear microsporidian parasites infecting marine fish and is robustly placed in the Enterocytozoonidae. Some features common to an intranuclear microsporidian previously reported from captive Canadian lumpfish are evident but DNA sequence data is required from Canadian lumpfish to confirm conspecificity.

Due to the importance of wild lumpfish fisheries in northern Atlantic countries, and their potential use as cleaner fish in Atlantic salmon farms, it is vital to evaluate this pathogen and its potential impact upon wild spawning stocks and captive fish.

## Competing interests

The authors declare that they have no competing interests.

## Authors’ contributions

AK performed the histological examination and drafted the manuscript; JMK sampled the fish, provided prevalence data and helped write the manuscript; MAF made the initial diagnosis, did the molecular study, developed the nested PCR, performed the phylogenetic analyses and helped write the manuscript. All authors approved the final version of the manuscript.
